# The virome of the kitome: small circular virus-like genomes in laboratory reagents

**DOI:** 10.1128/mra.01261-23

**Published:** 2024-04-09

**Authors:** Jiayi Duan, Emma Keeler, Alexander McFarland, Phillip Scott, Ronald G. Collman, Frederic D. Bushman

**Affiliations:** 1 Department of Microbiology, Perelman School of Medicine, University of Pennsylvania, Philadelphia, Pennsylvania, USA; 2 Department of Microbial Pathogenesis, Yale University School of Medicine, New Haven, Connecticut, USA; 3 Department of Pathobiology, School of Veterinary Medicine, University of Pennsylvania, Philadelphia, Pennsylvania, USA; 4 Pulmonary and Critical Care Division, Department of Medicine, Center for Translational Lung Biology, University of Pennsylvania School of Medicine, Philadelphia, Pennsylvania, USA; 5 Pulmonary and Critical Care Division, Department of Medicine, Lung Biology Institute, University of Pennsylvania School of Medicine, Philadelphia, Pennsylvania, USA; Katholieke Universiteit Leuven, Leuven, Belgium, USA

**Keywords:** virus, genome, virome, kitome, contamination

## Abstract

In the course of studying the virome of protozoan parasites, we identified small circular genomes resembling viruses, which turned out to be contaminants from an RNA purification kit. We report their sequences here so others can detect possible contamination in their samples by aligning them to these targets.

## ANNOUNCEMENT

We describe four small, circular virus-like sequences detected in laboratory reagents that we have provisionally named Amphisbainaviruses A–D. The name was inspired by Amphisbaina, a serpent from Greek mythology with a head at either end, which in this case refers to circularization via the detection of direct repeats on each end of linear virus-like contigs. These sequences were first identified in efforts to study viruses of *Leishmania* sp., but each was subsequently detected in H_2_O passed through RNA purification kits, documenting an origin in laboratory reagents.

Four different species of Leishmania parasites were cultured under standard axenic conditions (1–4) in Schneider’s medium supplemented with 20% FBS and 1% L-glutamine. One milliliter of supernatant from Leishmania culture or culture media-only control was centrifuged at 2,500 *g* for 10 min at 4°C, and the supernatant was passed through a 0.2 µm filter. The filtrate was concentrated using a 100 KDa-molecular-mass Amicon Ultra-15 centrifugal filter (Millipore, Cat No. UFC911024). The retained material was resuspended in 15 mL of SM buffer (50 mM Tris-HCl, pH 7.5, 100 mM NaCl, and 8 mM MgSO_4_), and the sample was concentrated to 500 µL and incubated with DNase I and RNase (Roche) at 37°C for 30 min. DNA and RNA were extracted from samples using an AllPrep DNA/RNA Mini Kit (Qiagen, Lot #172023856), and RNA was reverse transcribed into cDNA using a SuperScript III First-Strand Synthesis System Kit (Invitrogen). DNA and cDNA from the preparations were amplified by multiple displacement amplification (MDA) (5). Nextera XT (Illumina) libraries were prepared and sequenced with read lengths of 150 bp on the Illumina NextSeq 500, generating 4 million reads on average per sample. The Sunbeam pipeline was used (v3.1.1) for read quality control, adapter trimming, and contig assembly (6), and Cenote-Taker2 (7) was used to identify non-phage virus-like contigs (containing ≥1 viral hallmark) (5). Four small virus-like sequences were identified with complete circular genomes (Table 1).

**TABLE 1:** Genome sequences of Amphisbaina viruses^*a*^

Viral sequence	GenBank accession	BioProject	BioSample	SRA accession	Genome coverage (×)	Genome size (bp)	Genome GC content (%)	CDS annotation	Average virus copy number per mL based on qPCR	qPCR forward primer	qPCR reverse primer	qPCR probe	qPCR insert positive control plasmid
Amphisbainavirus-A	OR537218	PRJNA1012505	SAMN37261144	SRR25893174	455	909	42.2	Cap	180,319	CCAGGACGCCAGTAATCTTATAG	CCCTGGTAGCCTTCAACTTTAG	/56-FAM/ACGGAGCTT/ZEN/ACCGCGATGTATGAC/3IABkFQ/	CCAGGACGCCAGTAATCTTATAGAAGTCATACATCGCGGTAAGCTCCGTATAACCAGGCATATCGTTAACACTAAAGTTGAAGGCTACCAGGG
Amphisbainavirus-B	OR537220	PRJNA1012505	SAMN37261143	SRR25893175	601	934	39.7	Cap	14,045	ACTGCTGCTATCTGCGAATTTA	CCGCTACTGTCAACGAGTTATT	/56-FAM/TGGACAACA/ZEN/TGGTCGTTATGCTCC/3IABkFQ/	CTACCAGCTTTCGCTAGTTCTCTGGTTCTTTCCCACTTAGCTTTATATGCTGCACCACCTTTCTCGTGAGACTCCATAGGTAGTTCACCATACTCAACAAAGTCTCCGTCT
Amphisbainavirus-C	OR537221	PRJNA1012505	SAMN37261147	SRR25893165	1,873	936	40.8	Rep	11,735,882	AGACGGAGACTTTGTTGAGTATG	CTACCAGCTTTCGCTAGTTCTC	/56-FAM/TCACGAGAA/ZEN/AGGTGGTGCAGCATA/3IABkFQ/	AGACGGAGACTTTGTTGAGTATGGTGAACTACCTATGGAGTCTCACGAGAAAGGTGGTGCAGCATATAAAGCTAAGTGGGAAAGAACCAGAGAACTAGCGAAAGCTGGTAG
Amphisbainavirus-D	OR537222	PRJNA1012505	SAMN37261144	SRR25893174	153	1,838	49.9	Rep	1,022,340	GTTGTCCGCTCGTAGTGTAA	GCTCTGGTTCAGGAGATGATAG	/56-FAM/ACGATCCTT/ZEN/GAGCTGAGATTGCCG/3IABkFQ/	GTTGTCCGCTCGTAGTGTAAGATGGCATTGAGGGCACGATCCTTGAGCTGAGATTGCCGAATGTCCTTCTTGTTGGTATAAGACCATGAACAGGTCTCGCCTATCATCTCCTGAACCAGAGC

^*a*^ CDS, open reading frame is identified by ORFfinder (8) and then annotated by comparing to NCBI's non-redundant protein sequences database using BLASTp (9). Rep, replication-associated protein. Cap, capsid. Genome size, size of the viral contig identified. Genome GC content%, percent GC content of the viral contig identified. Information on qPCR amplicons: backbone for all positive control plasmids is pUC57. Sequence information of qPCR probes, primers, and positive controls is provided in the table.

To quantify the distribution of these sequences, we developed qPCR probes and primers (Integrated DNA Technologies; Table 1). Test qPCR reactions were run in triplicate. Positive controls were generated by synthesizing DNA plasmids with sequences matching the Amphisbainavirus amplicons (titrated from 10 to 10^8^ copies per PCR reaction). qPCR reactions were as follows: 95°C for 1 min, followed by 40 cycles of 3 seconds of 95°C plus 30 seconds of 60°C. The qPCR detection limit was 40 cycles.

To assess the source of Amphisbainaviruses, we passed water (Sigma-Aldrich, No. W4502-1L, Lot #BCCH9665) through the RNA purification kit (Qiagen AllPrep DNA/RNA Mini Kit Cat No. 80204, Lot #172023856; Qiagen RNeasy Plus Mini Kit Cat No. 74104, Lot #175011502) and then carried out the MDA amplification without including a reverse transcription step. After MDA amplification, viral genomes were detected by qPCR, ranging in abundance from 10^5^ to 10^7^ virus copies per mL (Table 1). We conclude that Amphisbainaviruses A–D are DNA contaminants introduced in the RNA purification kits.

This study thus expands the list of virus-like contigs contaminating metagenomic studies [e.g., (10–13)].

## Data Availability

See Table 1.
